# Supplementary material for: Assessment of chemical methods in the extraction of spore surface layers in Clostridioides difficile spores
Source: mSphere. 2025 Sep 15;10(10):e00531-25. doi: 10.1128/msphere.00531-25 (PMC12570504; doi:10.1128/msphere.00531-25)
Supplement: Figure S1 — Densitometric analysis of total protein from SDS-PAGE. [file msphere.00531-25-s0001.pdf]

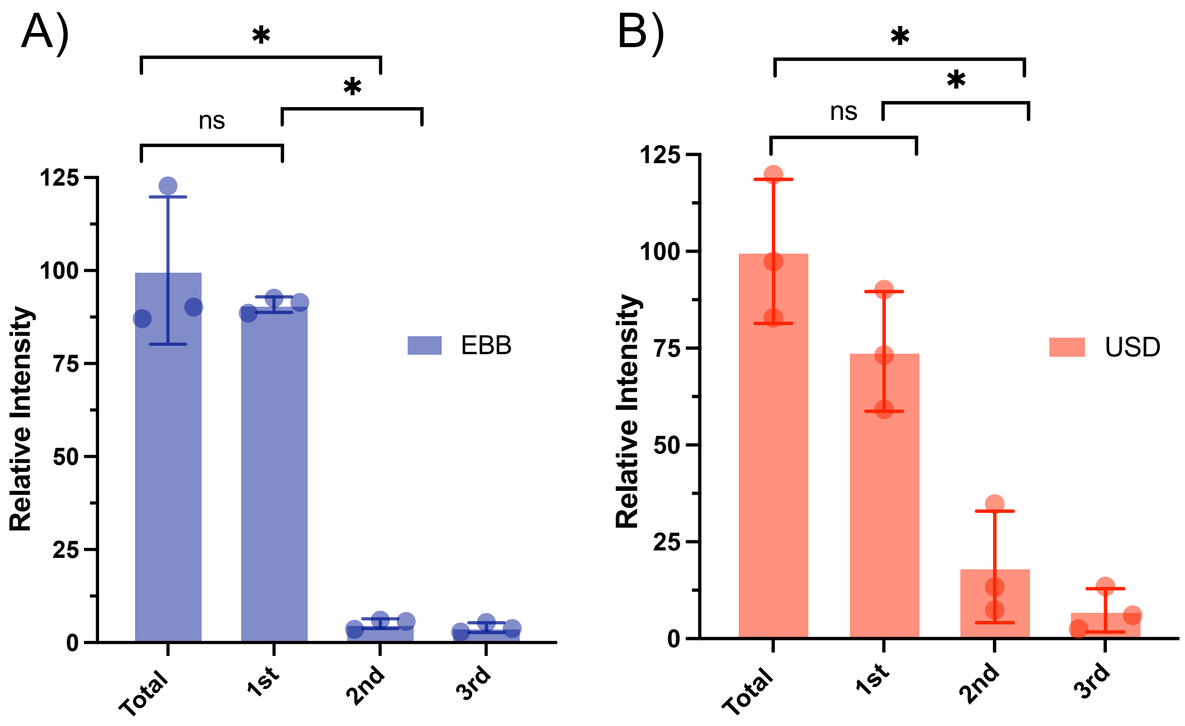

Figure S1. Densitometric analysis of total protein from SDS-PAGE

Band intensities were quantified by densitometry from SDS-PAGE gels, and normalized total protein for EBB and USD. Data represent the mean and standard deviation from three independent biological replicates. EBB treatment is colored in blue and USD red. Statistical analysis was performed using a one-way ANOVA to assess the effects of the respective extraction buffer EBB or USD and effect of continuous extractions on amount of protein removed. Statistical analysis post hoc comparisons were conducted using Tukey’s multiple comparisons test. Significance is indicated as ns (not significant), * $p < 0.05$.
