# Supplementary material for: Assessment of chemical methods in the extraction of spore surface layers in Clostridioides difficile spores
Source: mSphere. 2025 Sep 15;10(10):e00531-25. doi: 10.1128/msphere.00531-25 (PMC12570504; doi:10.1128/msphere.00531-25)
Supplement: Figure S2 — SDS-PAGE and immunoblots of purified soluble and insoluble CdeC and CdeM protein. [file msphere.00531-25-s0002.pdf]

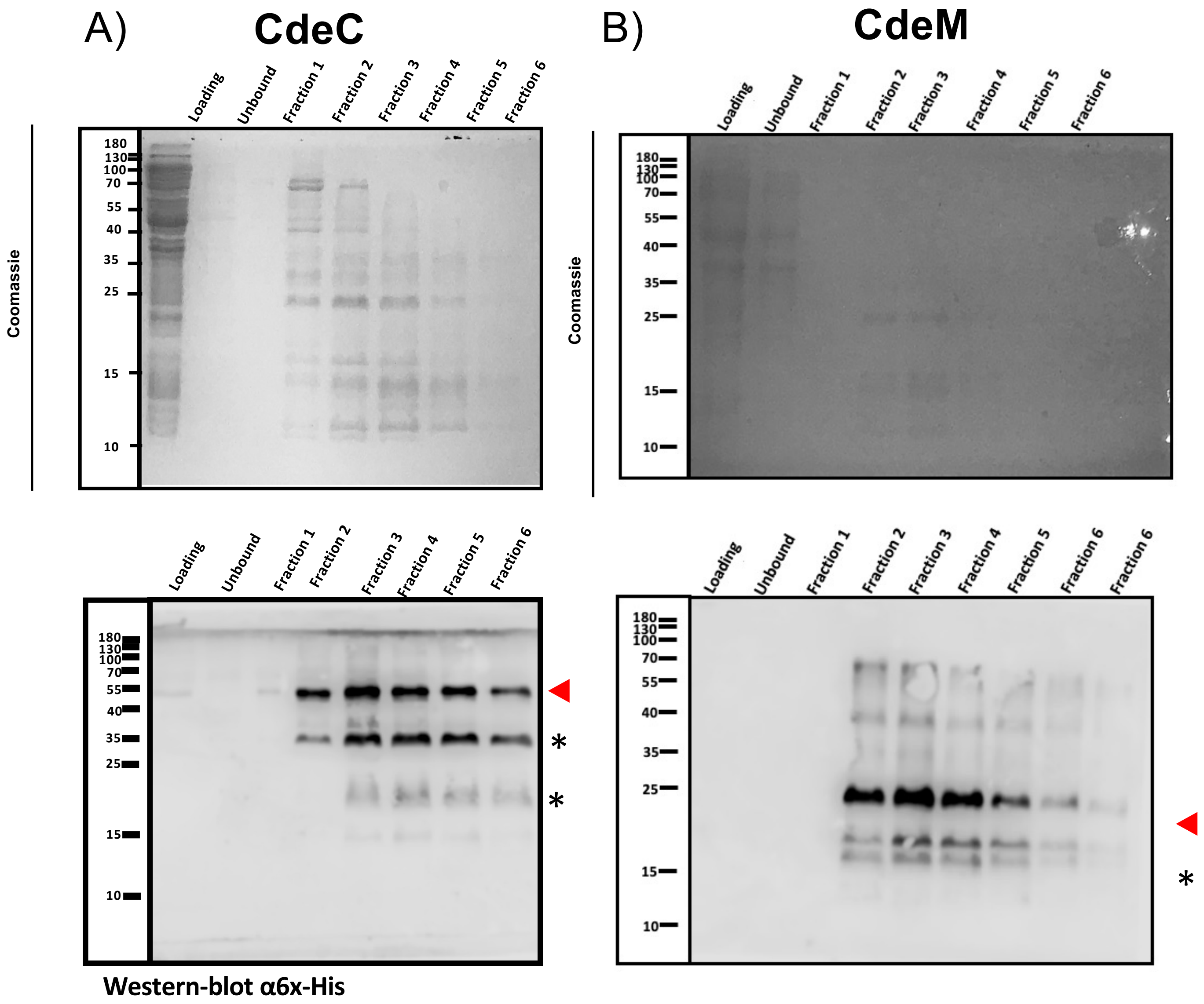

Figure S2: SDS-PAGE and Immunoblots of purified soluble and insoluble CdeC and CdeM protein

Purification of soluble recombinant CdeC and CdeM proteins was done using Akta Start, Cytiva FPLC protein purification system. Briefly, filtered soluble protein extract were loaded on HisTrap FF crude column (Loading) (GE Healthcare), washed with 15 column volumes (CV) of wash buffer (50 mM NaH_2PO_4, 300 mM NaCl, and 20 mM imidazole, pH=8). Proteins were eluted with 10 CV of elution buffer (50 mM NaH_2PO_4, 300 mM NaCl, and 250 mM imidazole, pH = 8) and marked as fractions 1-6. Samples were eluted as a single peak and subsequent fractions were analyzed by 15% SDS-PAGE and stained with Coomassie G250 to determine purity of fractions. Purified protein was blocked immunoblotted with mouse anti 6x His and incubated with goat anti mouse HRP. Immunoblot analysis revealed immunoreactive bands corresponding to monomeric form of CdeC and CdeM respectively are indicated by red arrows. CdeC has a predicted molecular weight of ~44 kDA whereas CdeM has a predicted weight of ~19.1 kDa. Asterisks indicate non-specific binding of antibody.
